# Bronchogenic Cyst Presenting as Acute Pericarditis

**DOI:** 10.7759/cureus.8874

**Published:** 2020-06-28

**Authors:** Karthik Ramireddy, Reshma R Golamari, Arun Minupuri, Shengnan Zheng, Jammie Menetrey

**Affiliations:** 1 Internal Medicine, Mercy Catholic Medical Center, Darby, USA; 2 Internal Medicine, Penn State Health Milton S. Hershey Medical Center, Hershey, USA; 3 Cardiology, Mercy Catholic Medical Center, Darby, USA

**Keywords:** bronchogenic cyst, pericarditis, mechanical impingement, pericardial effusion, mediastinum

## Abstract

Bronchogenic cysts are speculated to arise from abnormal budding of the foregut tissue during embryogenesis. Around 90% of mediastinal bronchogenic cysts are asymptomatic, and a small percentage of them present with chest pain and dyspnea. Pericardial effusion is one of the manifestations described; however, pericarditis has not been widely reported. We describe a case of a bronchogenic cyst in a 19-year-old female with an initial presentation of pericarditis due to mechanical impingement. There was an associated trace to small pericardial effusion. The bronchogenic cyst was planned to be excised; however, it could only be partially excised due to its adherence to the left atrium.

## Introduction

Bronchogenic cysts are thin-walled, fluid- or mucus-filled cysts that result from anomalous development of the ventral foregut. They are most often found in the mediastinum or lung [1], with a predilection for the carinal region, but sometimes can occur in remote sites such as intracardiac locations [2] or neck [3]. Even though they are relatively rare, they represent the most common cystic lesions of the mediastinum and account for nearly 6-15% of all mediastinal tumors [4]. More than 90% of patients with bronchogenic cysts are asymptomatic and are usually diagnosed as incidental radiological findings [5]. We describe a case of bronchogenic cyst that presented as pericarditis due to mechanical stress applied by the cyst on the heart.

## Case presentation

A 19-year-old Caucasian female with no medical history presented with chest pain, substernal in location that was radiating to her back. She described the pain as worsening when lying flat and with deep inspiration, with improvement on sitting upright. It was associated with shortness of breath, nausea, and vomiting. Her social history was significant for tobacco use of half a pack for two years and occasional marijuana use.

**Figure 1:**
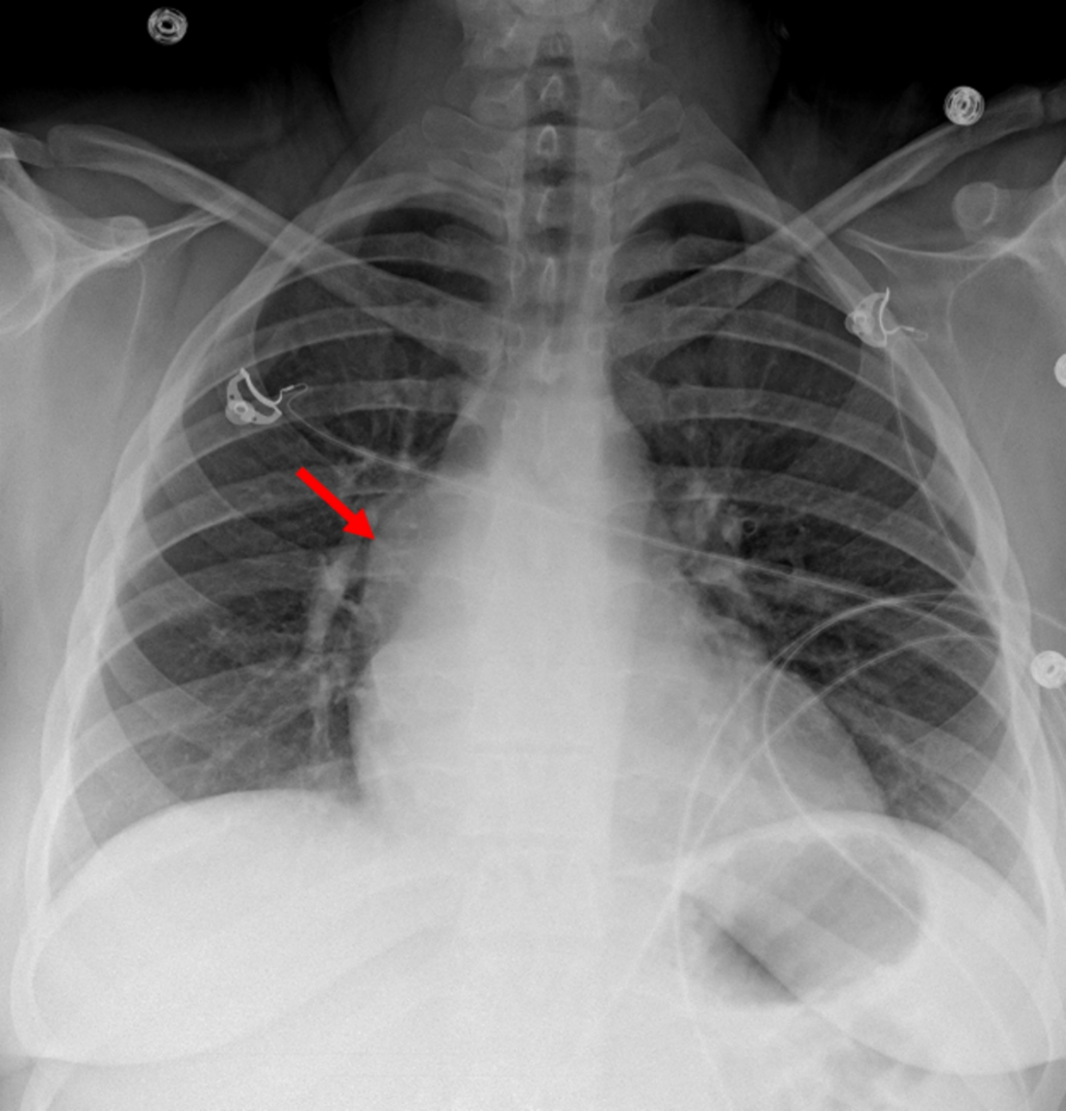
Chest X-ray (posteroanterior view) showing abnormal bulging of the right mediastinal contour (red arrow), suggestive of a mediastinal mass.

**Figure 2:**
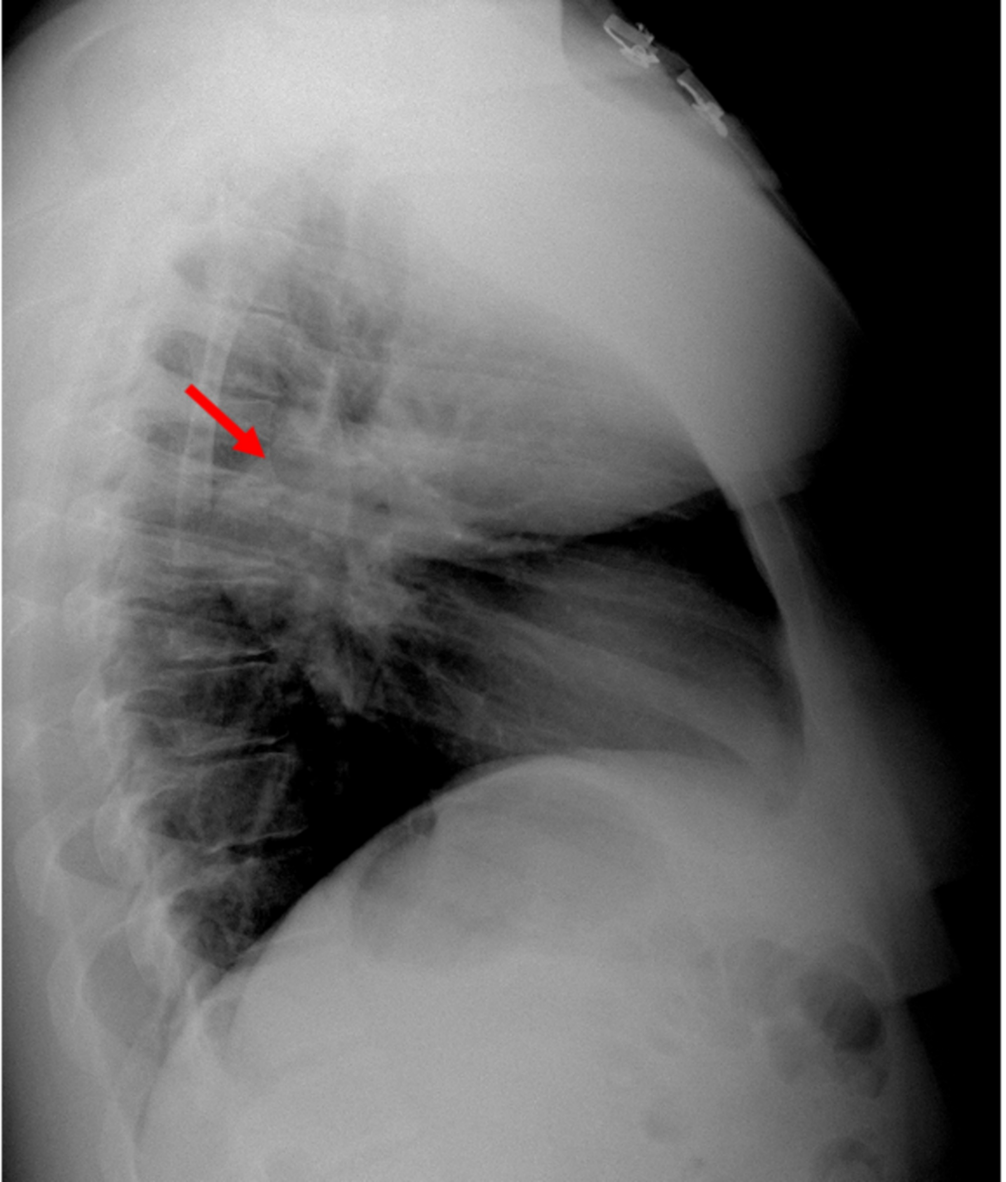
Chest X-ray (lateral view) showing a mass-like lesion in the posterior mediastinum (red arrow).

**Figure 3:**
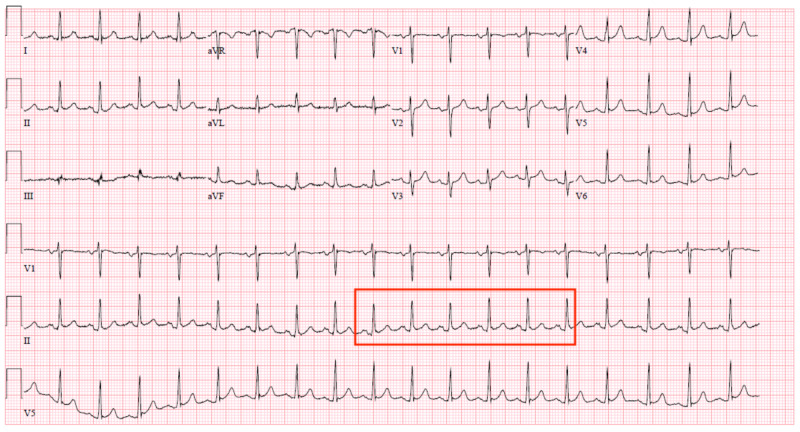
Electrocardiogram showing sinus tachycardia.

**Figure 4:**
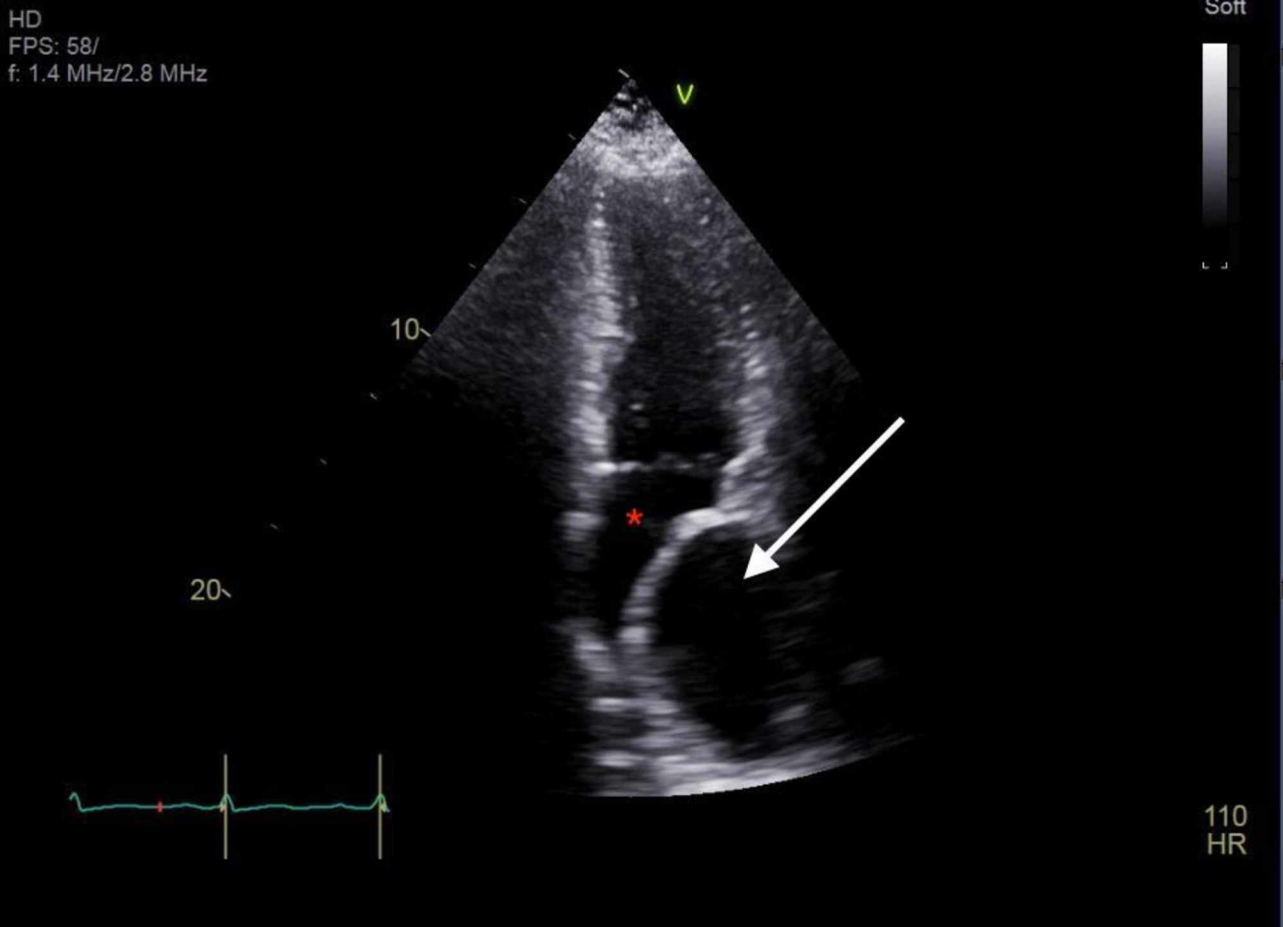
Transthoracic echocardiogram showing cyst-like structure (white arrow) compressing the left atrium (red asterisk).

**Figure 5:**
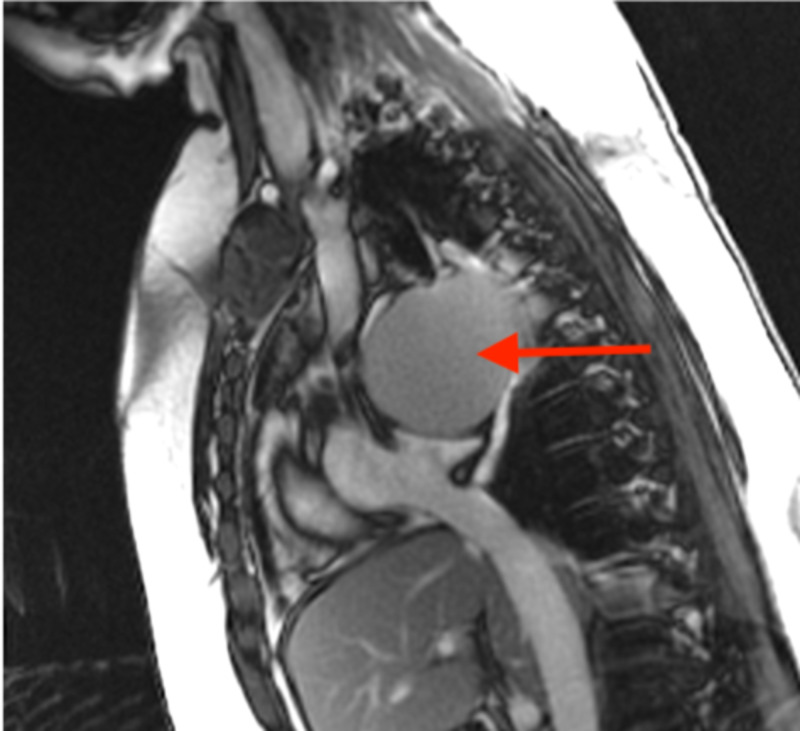
Cardiac MRI (FIESTA, RVOT view) showing an 8.2-cm subcarinal cystic lesion (red arrow) with uniform T2 hyperintensity, exerting mass effect on the left atrium. No large solid component is identified. FIESTA, fast imaging employing steady-state acquisition; RVOT, right ventricular outflow tract

**Figure 6:**
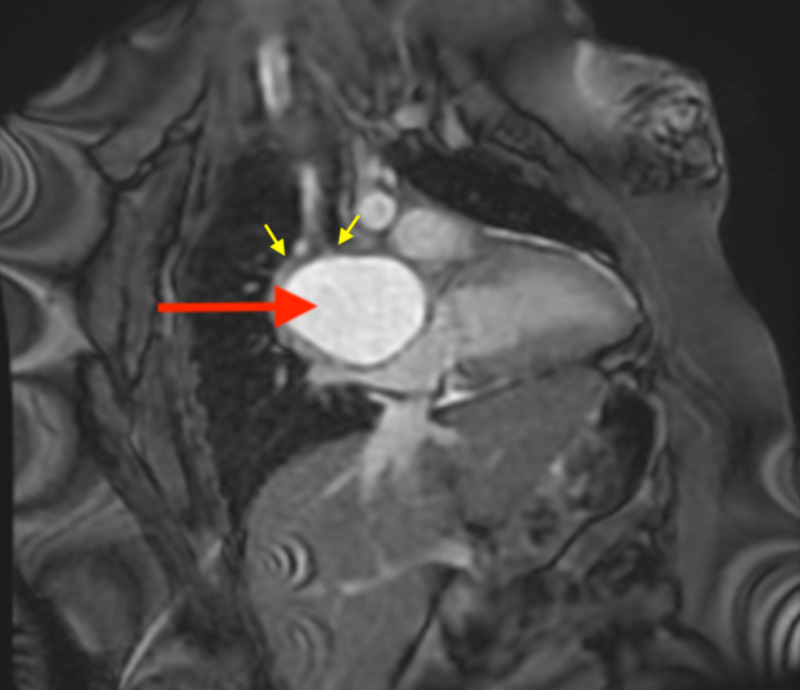
Cardiac MRI (FIESTA, fat-saturated two-chamber view) showing T2 hyperintense mediastinal mass (red arrow), splaying the mainstem bronchi (yellow arrows). FIESTA, fast imaging employing steady-state acquisition

**Figure 7:**
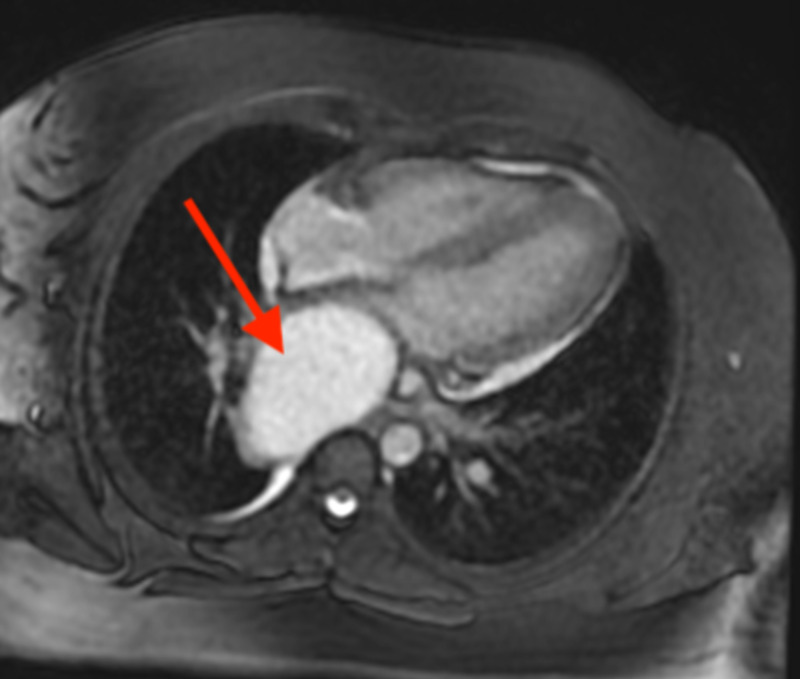
Cardiac MRI (FIESTA, fat-saturated axial four-chamber view) showing uniformly T2 hyperintense retrocardiac mass (red arrow). FIESTA, fast imaging employing steady-state acquisition

At presentation, the chest X-ray showed abnormal bulging of the right mediastinal contour, suggestive of a mediastinal mass (Figures 1, 2). The electrocardiogram showed sinus tachycardia (Figure 3). Three sets of troponin and a D-dimer level were negative. Her erythrocyte sedimentation rate (ESR) and C-reactive protein (CRP) level were elevated at 38 mm/hour and 33.2 mg/L, respectively. A transthoracic echocardiogram was performed, which showed a trace to a small amount of pericardial effusion without evidence of pericardial tamponade. It also showed a cyst-like structure near the left atrium (Figure 4). Further tests included cardiac magnetic resonance imaging (MRI), which showed a subcarinal mass measuring up to 5.6 cm x 8.2 cm x 6 cm with uniform T2 hyperintensity and was interpreted as a probable bronchogenic cyst. This cystic lesion exerted a mass effect on the left atrium (Figures 5-7).

**Figure 8:**
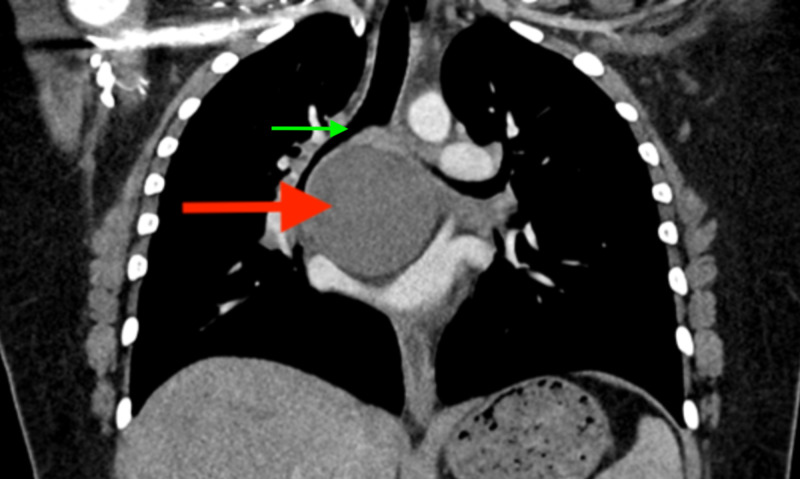
Chest CT (coronal view) with intravenous contrast showing thin-walled cyst (red arrow) in the subcarinal region with internal minimally complex fluid density. The cyst splays the right and left mainstem bronchus (green arrow).

**Figure 9:**
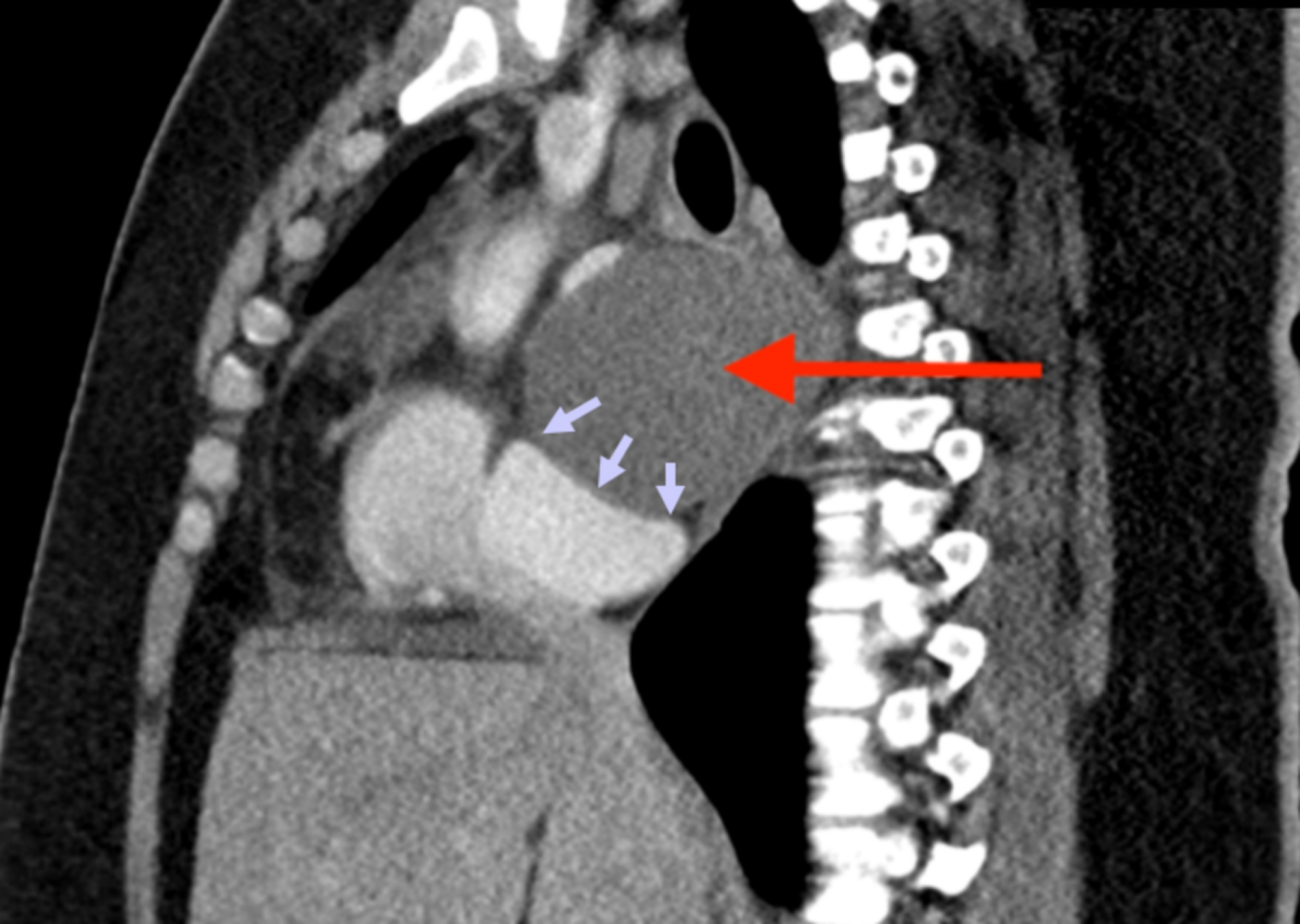
Chest CT (sagittal view) with intravenous contrast showing fluid-filled subcarinal mass (red arrow), causing a mass effect upon the left ventricle (purple arrows).

**Figure 10:**
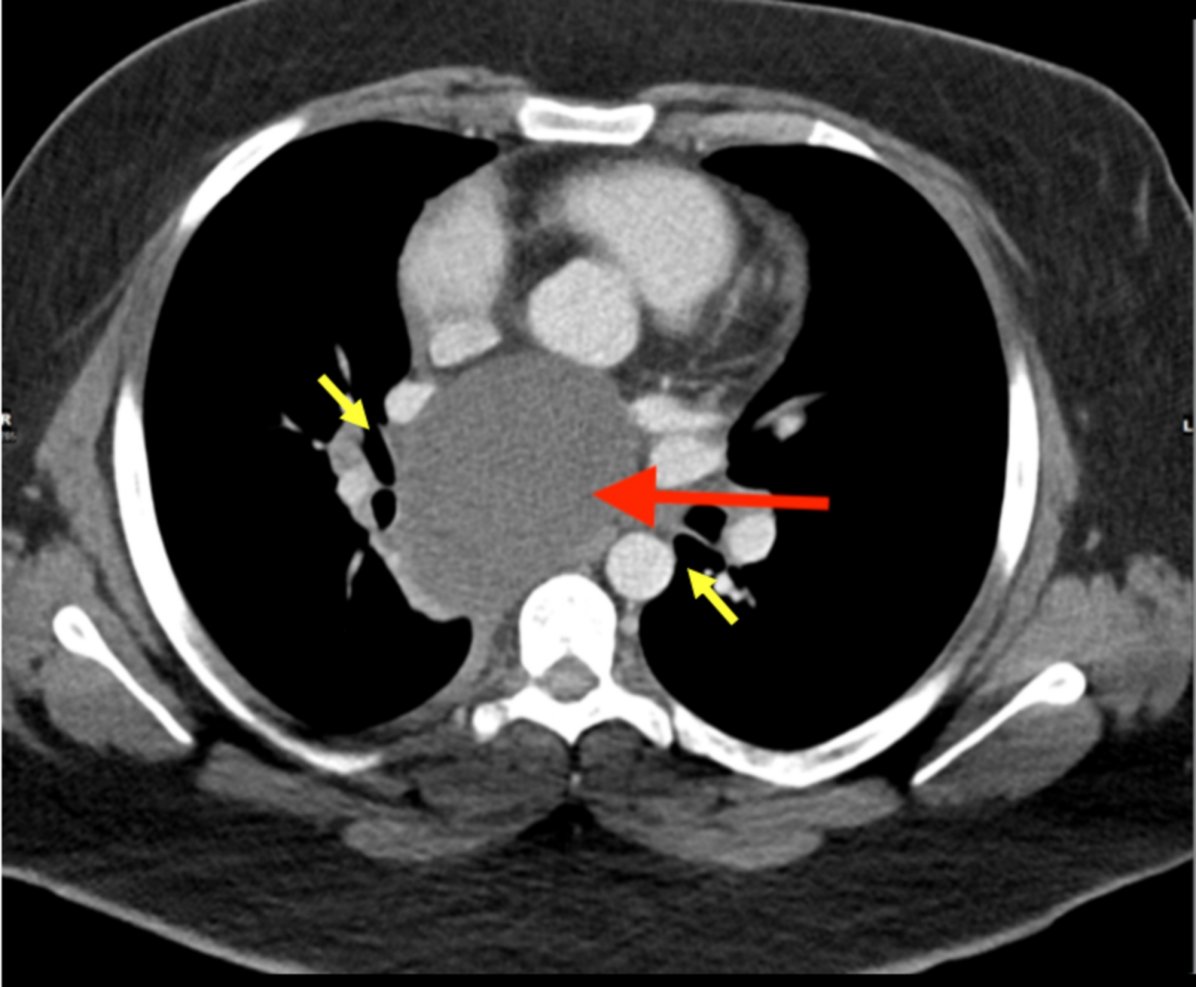
Chest CT (axial view) with intravenous contrast showing a 7.5 x 6.8 x 6.3 cm mildly complex fluid-filled thin-walled subcarinal mass (red arrow). The mass is splaying the right and left mainstem bronchi (yellow arrows).

The patient was immediately started on a high dose of ibuprofen at 600 mg three times a day, colchicine 0.6 mg twice a day, and pantoprazole sodium 40 mg twice a day before meals for clinical suspicion of pericarditis. This intervention improved her symptoms within two days. Cardiothoracic surgery service was consulted, and a computed tomography (CT) chest with intravenous contrast was performed to better delineate the bronchogenic cyst morphology. It showed a 7.5 cm x 6.8 cm x 6.3 cm fluid-filled thin-walled subcarinal mass consistent with a bronchogenic cyst (Figures 8-10). Subsequently, she underwent right-sided video-assisted thorascopic surgery (VATS), and a large bronchogenic cyst, significantly adherent to its surrounding structures, was seen. Subsequently, a decision was made to convert the surgery into a thoracotomy to facilitate better visualization. Manual palpation demonstrated a large cyst adherent to the esophagus, left atrium, carina, and middle and lower lobes of the right lung. The cyst was then decompressed with a spinal needle and fluid was sent for cytology and gram staining. Complete removal of the cyst was not attainable due to its adherence to the left atrium. Therefore, only 50% of the cyst was successfully removed and a right 28-French pericardial drain was placed. She was transferred to the intensive care unit postoperatively, where she received adequate pain control along with incentive spirometry therapy. The chest tubes were removed three days later. The fluid from the cyst revealed no white blood cells or microorganisms on microscopy, and culture did not demonstrate any growth after three days. Histological analysis revealed a hemorrhagic and inflamed cyst wall lined by benign ciliated columnar epithelium, confirming the diagnosis of a bronchogenic cyst. The patient had continued improvement in her symptoms during her hospital stay and was discharged home on ibuprofen, pantoprazole, and colchicine.

**Figure 11:**
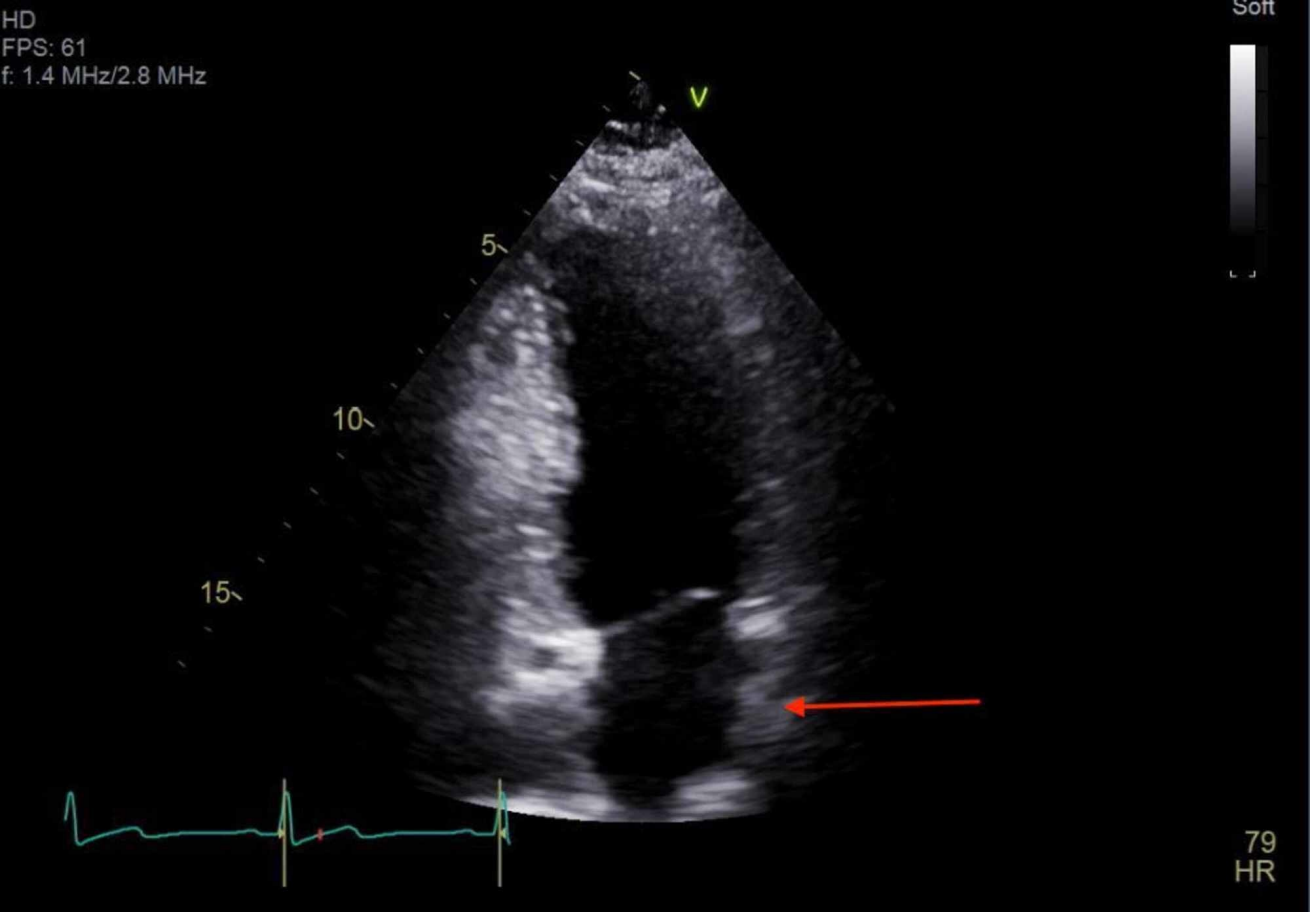
Transthoracic echocardiogram repeated one month later showed thickening (red arrow) near the left atrial wall due to known remnants of the bronchogenic cyst.

Upon follow up one month later, a repeat echocardiogram was performed, which revealed a trace to a small amount of pericardial effusion without evidence of tamponade, and an evidence of thickening near the left atrial wall due to known remnants of the bronchogenic cyst (Figure 11).

## Discussion

Bronchogenic cysts commonly arise in the mediastinum due to abnormal budding of the ventral diverticulum of the foregut between the 26th and 40th days of intrauterine life [6]. They can arise in the lungs, diaphragm, retroperitoneum, pericardium, thymus, or unusually in the head/neck [7]. In the mediastinum, the cysts can be mainly found adherent to the esophagus, tracheobronchial tree, pericardium, and lungs [1]. Structurally they are unilocular cysts containing clear fluid, hemorrhagic secretions, or air [8], and are lined by ciliated epithelium, with the inclusion of hyaline cartilage, smooth muscle, and bronchial gland [1].

Clinical manifestation may range from being asymptomatic to compression, infection, pneumothorax, hemorrhage, or rupture [9]. If symptomatic, they usually present as back pain or cough [10]. Other symptoms include chest pain, abdominal pain, and compressive symptoms of dyspnea and dysphagia. Cardiac arrhythmias [11] and, in rare cases, fistulization of the airway has also been reported [1].

Bronchogenic cysts are filled with fluid [12] containing a mixture of water and proteinaceous mucus [13]. A chest X-ray may be useful in identifying a majority of them [14] and is particularly useful when they have an air-fluid level. CT chest is another means of diagnosis, and the cysts appear as homogenous soft tissue hypoenhancing mass(es) [10] that are oval/spherical with smooth outlines. Calcification may be observed in 10% of the patients [15]. MRI of the chest may also be used to diagnose these lesions, which is superior to CT in attaining an accurate preoperative diagnosis [16]. Even though imaging modalities can characterize the lesion, surgical excision with histopathological examination remains the mainstay for a definitive diagnosis.

Differential diagnoses of a bronchogenic cyst include gastrointestinal duplication cyst, cystic pulmonary sequestration, cystic teratoma, mesothelium-lined cyst, posttraumatic cyst, and hydatid cyst [15].

For symptomatic patients, treatment is usually by complete resection which prevents complications, whereas in asymptomatic patients, there is some controversy regarding resection. Studies show that approximately 45% of asymptomatic cysts at diagnosis, if followed over time, develop symptoms and have a 0.7% risk of malignant transformation [17]. Based on this observation, many surgeons recommend surgery, even though the surgery itself carries a peri-operative risk of 20% morbidity [17]. On the other hand, delaying surgery with close follow-up until the development of symptoms is also considered an acceptable approach.

The type of surgical approach is decided on a case-to-case basis. Open thoracotomy is preferred over VATS in the setting of major adhesions to vital structures. Even when performing a thoracotomy, complete excision maybe not feasible at times in order to avoid structural damage to the involved organs. In these situations, subtotal resection is performed along with cauterization [18].

## Conclusions

The majority of the bronchogenic cysts are asymptomatic and discovered incidentally on routine imaging studies. Symptoms arise when the cyst gets infected or if there is any tracheobronchial or cardiac compression. As there is high variability in their location, the presentation of the symptomatic bronchogenic cysts is highly inconsistent. Various imaging modalities are available to obtain a pre-operative diagnosis, including chest X-ray, chest CT scan, and cardiac MRI. Surgical excision is both diagnostic and potentially curative and is recommended even in asymptomatic cases to avoid the development of future complications including malignant transformation.
